# REACHING THE LIMIT: CENTENARIANS IN THE HEALTH AND RETIREMENT STUDY

**DOI:** 10.1093/geroni/igac059.1202

**Published:** 2022-12-20

**Authors:** Peter Martin, Jennifer Ailshire

## Abstract

Although life expectancy has increased significantly over the last century, it is still unlikely that individuals reach the century mark of their lives. As a result, it is difficult to study a large enough sample of centenarian survivors; it is even more difficult to follow developmental trajectories of those who survive into very late life. The AHEAD sample of the Health and Retirement Study (HRS) contains longitudinal data of older adults who first participated in 1993. More than 500 HRS participants survived to at least 98 years of age. In this symposium, we present three uses of the data: first, we compare centenarians to older adults who did not survive into their nineties. Second, we compare different cohorts of centenarians with regard to health and psychosocial behavior. Third, we follow participants from their eighties to 100 years of age. The first presentation provides an overview of the HRS subsample. The second presentation highlights the personality profiles of centenarians. The third presentation traces health and psychological well-being among centenarians in the HRS. Finally, we discuss trajectories of cognition and functional limitations for three cohorts of centenarians. The results provide important information for policies and practical implications for families and service providers to older adults, highlighting available resources and health and well-being changes in very late life.

